# Beyond comprehensible input: a neuro-ecological critique of Krashen's hypothesis in language education

**DOI:** 10.3389/fpsyg.2025.1636777

**Published:** 2025-10-17

**Authors:** Quang Nhat Nguyen, Dung Thi Hue Doan

**Affiliations:** Saigon International University, Ho Chi Minh City, Vietnam

**Keywords:** comprehensible input, neuro-ecological approach, affordance theory, adaptive learning, AI in education, language learning

## Abstract

This article critically reassesses the Comprehensible Input (CI) hypothesis in language education by drawing on recent advances in neurolinguistics and an ecological perspective on learning. While the CI hypothesis claims that language is acquired by understanding input slightly beyond a learner's current competence (i+1), converging evidence from brain research shows that language development is an active and embodied process supported by interaction, feedback, and multimodal engagement. From the ecological point of view, affordances are the perceivable opportunities for action that arise in the ongoing coupling of learner and environment. Using this combined neuro-ecological lens, the paper critically reviews empirical studies from the last three decades and demonstrates that meaningful language growth depends on learners detecting and acting on such affordances rather than merely processing linear and simplified input. Adaptive and AI-supported learning systems further illustrate how contemporary technologies can operate these mechanisms and offer individualized, scalable alternatives to the static i+1 model. The analysis argues that CI should no longer serve as a central doctrine in language education and calls for pedagogies that reflect the interactive, affordance-rich processes revealed by current brain and language science.

## Introduction

A new wealth of interdisciplinary research in neurolinguistics, brain-based learning, and ecological language perspectives has cast fresh doubt on the comprehensible input hypothesis. Stephen Krashen's Comprehensible Input (CI) hypothesis has historically held sway in Second Language Acquisition (SLA) theory and pedagogy. Krashen (1982) famously argued that learners acquire language “in only one way”, by understanding input that is just beyond their current level of competence (the i+1 principle; Krashen, 1982). Introduced as part of his Monitor Model in the late 1970s and 1980s, this hypothesis shifted language teaching toward meaning-focused exposure and away from rote grammar drills (Krashen, 1985). Indeed, Krashen's ideas spurred approaches like the Natural Approach and Communicative Language Teaching, emphasizing immersive exposure over explicit instruction (Krashen and Terrell, 1983). Historically, the input hypothesis had a profound impact, becoming “the single most important concept in SLA” in Krashen's view (Krashen, 1996) and attracting many practitioners worldwide (Lightbown, 2019). However, from its inception, the theory was also highly controversial. Critics argued that Krashen's claims were vague, untestable, and overstated, lacking clear definitions and robust empirical support (McLaughlin, 1978; Gregg, 1984; Gass and Selinker, 2008).

Findings from cognitive neuroscience show that language learning is not a passive process of absorbing input, but an active, embodied one, engaging multiple brain systems through interaction, feedback, and multimodal experience (Pulvermüller, 2018; Tomasello, 2019). Likewise, ecological theories (e.g., Van Lier's affordance model) reconceptualize language learning as emerging from learners' dynamic interactions with rich environments, rather than from simplified, teacher-controlled input (van Lier, 2004; Larsen-Freeman, 2018). In parallel, advances in personalized and AI-supported learning demonstrate that a one-size-fits-all i+1 approach fails to address individual learner pathways, whereas adaptive systems can finely tune input and practice to each learner's needs (Nguyen, 2022a; Paladines and Ramirez, 2020; Lin et al., 2023). In short, Krashen's comprehensible input hypothesis appears conceptually flawed, empirically outdated, and practically insufficient in modern contexts.

This article systematically critiques the CI hypothesis through an interdisciplinary lens. We begin by outlining Krashen's original hypothesis and its legacy. We then synthesize evidence from neurolinguistics and brain research showing that language acquisition is an active, neuroplastic process that depends on interaction and embodied experience rather than the passive consumption of input. Next, we draw on an ecological framework, including van Lier (2004) and Nguyen's (2022a,b, 2025a,b) work on affordance theory, to argue that meaningful learning emerges when learners engage with rich, multimodal environments instead of relying on simplified input alone. Within this integrated neuro-ecological view, experience-dependent brain plasticity operates in tandem with the ecological processing of affordances, allowing linguistic knowledge to continually emerge and reorganize as learners act within their environments. We critically examine the i+1 concept's limitations in scaling and personalization, contrasting it with modern individualized, data-driven learning pathways and AI-enhanced environments. A detailed neuro-ecological review of recent empirical studies in neurolinguistics, ecological language learning, and adaptive learning supports this critique. We conclude by calling for the abandonment of CI as a guiding pedagogical doctrine and for a reconceptualization of language education using neuro-ecological and affordance-based frameworks that better reflect how languages are learned in the twenty first century.

## Krashen's comprehensible input hypothesis: theory and impact on language education

Stephen Krashen's Input Hypothesis emerged in the late 1970s as part of his broader Monitor Model of SLA. The Input Hypothesis posits that “language is acquired by receiving comprehensible input slightly above one's current level of competence (i+1)” (Krashen, 1982, p. 21). In practical terms, if a learner's proficiency is *i*, then exposure to language just one step beyond that level (i+1) will spur acquisition, provided the input is understood, made comprehensible through context, or aided. Krashen maintained that acquisition is distinct from conscious learning: given sufficient comprehensible input and low anxiety (a low affective filter), learners will subconsciously absorb the new language (Krashen, 1985). Grammar teaching, error correction, or forced output were deemed unnecessary or secondary; understanding input was considered necessary and sufficient for acquisition in most cases (Krashen, 1985, 1996). This stance was encapsulated in Krashen's bold claim that comprehensible input is the causal factor in SLA, and that “all other factors… work only when they contribute to comprehensible input and/or a low affective filter” (Krashen, 1985, p. 4). In other words, motivation, interaction, or output matter only insofar as they increase understandable input or reduce anxiety, according to Krashen.

The impact of this hypothesis on language education was far-reaching. Krashen's ideas helped catalyze a paradigm shift in the 1980s from grammar-intensive methods (e.g., audiolingual drills, grammar-translation) to more meaning-focused, communicative approaches (Lightbown, 2019). Along with Terrell, Krashen developed the Natural Approach, encouraging teachers to flood students with slightly challenging input in low-anxiety settings, postponing speaking until learners felt ready (Krashen and Terrell, 1983). While rooted in broader sociolinguistic theory, the Communicative Language Teaching (CLT) movement found a strong theoretical justification in the Input Hypothesis' emphasis on meaningful exposure over explicit grammar instruction (Long, 1996; Richards and Rodgers, 2014). Teachers were encouraged to use simplified target language, visuals, and gestures to make input comprehensible, assuming that if students understood the message, even with some new structures present, their language proficiency would naturally grow. This approach appealed to practitioners for its intuitiveness (mirroring how children seem to pick up language) and its humane, low-stress ethos. Krashen often invoked the example of children acquiring L1 through immersion in language they only partly understand, not through grammar drills. By the late twentieth century, the comprehensible input principle had become attractive to many language teachers worldwide, profoundly influencing curricula, teacher training, and even popular self-study methods. Despite, or perhaps because of, its popularity, Krashen's theory quickly attracted extensive criticism. Scholars noted that the Monitor Model, including the Input Hypothesis, was “one of the most controversial theoretical perspectives in SLA in the last quarter of the twentieth century” (Gass and Selinker, 2008, p. 298).

## Insights from neurolinguistics neurolinguistic evidence: language learning as an embodied, interactive, and neuroplastic process

In contrast to Krashen's theory, modern neurolinguistic research shows that language development is not a passive process of absorbing comprehensible input. Instead, it is a constantly active, brain-modifying endeavor that requires sensorimotor engagement, social interaction, and continual feedback. Krashen's hypothesis, by contrast, depicts the learner's mind as a language “acquisition device” that merely needs understandable input and low anxiety to operate (Krashen, 1982). Contemporary cognitive neuroscience paints a very different picture: understanding language is inseparable from using language, and neuroplastic change is driven as much by practice and embodied experience as by exposure itself (Pulvermüller, 2018; Li and Jeong, 2020; Tomasello, 2019).

First, consider the role of neuroplasticity and active engagement. Neuroplasticity refers to the brain's ability to rewire by forming new neural connections in response to learning and practice. To induce such changes, mere exposure is often insufficient; active processing and retrieval of language seem to be critical (Pulvermüller, 2018; Ellis, 2008). Studies have shown, for example, that when learners actively produce language or engage in conversation, distinct brain networks are recruited compared to when they only comprehend input. In a meta-analysis of neuroimaging studies, language production (speaking) was associated with additional activation in right-hemisphere frontal and temporal regions related to motor planning, whereas comprehension primarily engaged left-hemisphere language regions (Indefrey and Levelt, 2004; Price, 2012). In other words, producing language uses broader neural circuitry than listening, engaging areas involved in motor control, sensory feedback, and cognitive monitoring. This suggests that active use provides a different and arguably deeper kind of learning stimulus to the brain than passive input; it lights up more of the brain, creating more pathways that can later consolidate into new language skills (Pulvermüller, 2018).

Additionally, brain-based research on embodied cognition finds that understanding language often involves simulating experiences; for instance, hearing action verbs can activate motor cortex regions as if the listener were performing the action (Pulvermüller, 2018; Barsalou, 2008). Such findings align with the idea that language is grounded in sensorimotor and emotional systems (Tomasello, 2019). If language meaning is tied to perception and action, learning words and structures might require connecting them to embodied experiences, not just hearing them in simplified sentences. A purely input-focused approach might under-stimulate these essential brain processes (Gullberg, 2010). Verbotonalism, as discussed in Yang et al., 2023 and Cai et al. (2021), highlights the integral role of prosody, particularly intonation, in the perception and production of individual sounds. According to Cai et al. (2021), intonation is ever-present in spoken language and influences the quality of vowels and consonants, suggesting that focusing on prosodic features can enhance pronunciation more effectively than segmental approaches alone.

Crucially, social interaction and feedback have been shown to enhance neural and cognitive outcomes in language learning, beyond what input alone can achieve. Recent works on what has been called the “social brain of language” emphasize that humans are biologically tuned to learn more effectively, but not exclusively, through interaction, though learning is still possible through other mechanisms. Li and Jeong (2020) review evidence that language learning “unfolds in socially interactive contexts” and report that “work from several recent L2 studies also suggests positive brain changes along with enhanced behavioral outcomes as a result of social learning” (p. 1). These positive brain changes refer to neuroplastic adaptations, such as growth in neural connectivity or more efficient brain activation patterns, which are observed when learners engage in communicative interaction in the L2. One illustrative study found that adult learners who practiced conversation with feedback showed greater increases in brain activity related to syntactic processing (and made larger proficiency gains) than those who spent equivalent time passively watching L2 videos (Li and Jeong, 2020). Interactive conditions likely stimulate neurotransmitter systems (e.g., dopamine release associated with reward and motivation) that facilitate memory and learning (Ullman, 2016). Indeed, neuroscience has demonstrated that novelty and active involvement trigger dopamine in the brain's reward pathways, strengthening new information retention (Lisman et al., 2011). A classroom solely centered on teacher-provided comprehensible input, especially if it involves little novelty or student agency, risks lower engagement on a neurological level. By contrast, a class that prompts learners to interact, solve problems, and get immediate feedback on their output will activate attentional and reward networks that enhance learning and memory consolidation. Feedback closes the loop for the brain: it allows the learner to correct errors and adjust their internal representations. Brain imaging research on second-language training has shown associations between corrective feedback and activation in prefrontal and language-related regions, consistent with the idea that the brain updates linguistic representations during feedback-based learning (Meyer et al., 2013; Li, 2010). These interactive, feedback-driven processes are not accounted for in Krashen's input-centric model, where understanding input alone is meant to suffice. Modern neurolinguistics instead indicates that language acquisition is an active skill acquisition process, more analogous to learning a musical instrument or sport than to filling an empty vessel with content. As with those domains, practice (including output) and feedback are key drivers of improvement, physically shaping neural pathways over time (Pulvermüller, 2018; Ullman, 2016).

Another line of evidence comes from embodied language learning and multisensory integration. A growing body of research shows that pairing language with sensory, motor, or emotional experiences can significantly boost acquisition (Gullberg, 2010; Macedonia and Von Kriegstein, 2012). For example, young learners who gesture or act out meanings while learning new vocabulary tend to remember those words better, and neuroimaging confirms that their sensorimotor brain regions become linked to the word representations (Macedonia and Von Kriegstein, 2012; Ping et al., 2014). A review of embodied learning in language education identified that embodied approaches (from drama and movement activities to VR immersion) improve language skills and increase emotional and motivational engagement, providing a more holistic learning experience for students (Macedonia and Von Kriegstein, 2012). Such findings underscore that language is learned and stored in the brain alongside a rich tapestry of sensory and contextual information, not as isolated linguistic code. Comprehensible input, especially when “finely tuned” to be just a bit above the current level, often involves simplifying and decontextualizing language (Ellis, 2008). This contradicts what we know about memory: information with multiple modalities and rich context is remembered far better than bare-bones input. The CI hypothesis also largely ignores the affective dimension (beyond “keep anxiety low”), but brain research indicates that emotional resonance and learner investment in content can drive attention and memory (Tomasello, 2019).

In short, brain-based evidence rejects the notion of the learner as a passive sponge for input. Instead, it portrays learners as active meaning-makers whose brains thrive on interaction, multisensory context, emotional engagement, and producing language. An input hypothesis that does not incorporate these active ingredients is neurologically implausible. Understanding language is necessary, of course, but understanding alone, without use or contextual grounding, is not sufficient to rewire the brain's linguistic capacities (Nguyen, 2022b). As (Gregg 1984) noted, Krashen offered no mechanism for converting comprehended input into acquired competence. Modern neuroscience suggests that the mechanisms involve practice, feedback, and embodied experience, triggering neuroplastic changes, factors largely outside the scope of the original Input Hypothesis.

## Ecological theory and affordances: context, interaction, and multimodality in language education

While neurolinguistics shines a light on internal brain processes and the importance of action for learning, ecological approaches to language learning shift the focus to the external environment, the whole context in which learning occurs. An ecological perspective, as articulated by scholars like van Lier (2000, 2004), posits that language acquisition emerges from the relationship between learners and their environment, not from input *per se*. The environment offers affordances for learning, opportunities for meaningful action and perception, and learners actively pick up on these affordances to develop language (van Lier, 2000, 2004). This starkly contrasts with the Comprehensible Input (CI) model in which the environment is essentially a delivery mechanism for graded input. Here, we explore how affordance theory and ecological thinking expose fundamental shortcomings in the comprehensible input concept, arguing that rich, multimodal interaction in context is the real driver of language development.

Leo Van Lier famously suggested the field move “from input to affordance” in understanding language learning (van Lier, 2000, 2004). Borrowing from Gibson's (1979) ecological psychology, an affordance is defined as a “reciprocal relationship between an organism and a particular feature of its environment” (van Lier, 2004, p. 5; Gibson, 1979). The environment has various properties that can afford (i.e., enable or invite) specific actions to an active organism. For example, a tree affords climbing to a child, shade to a picnicker, or food to a caterpillar (Gibson, 1979; van Lier, 2004). For instance, a billboard in a target language is an affordance: it offers written language input, visual context, and perhaps cultural insight. A conversation with a native speaker is a rich affordance: it offers comprehensible input, but also the chance to negotiate meaning, to gesture, to observe social cues, etc. Crucially, affordances do not cause learning automatically, just as a tree does not cause a child to climb, but they invite active engagement. The learner must perceive and act upon the affordance for learning to occur (van Lier, 2000, 2004). Van Lier succinctly said, “Ask not what's inside your head, ask what your head's inside of” (van Lier, 2000, p. 6).

Whether something becomes an affordance depends on the organism's capabilities and interests. In the context of language, an affordance is a property of the meaningful or useful environment for communication, relative to the learner's goals and abilities (van Lier, 2004). Building on van Lier's (2000) call to move “from input to affordance,” a growing body of scholarship now treats affordance as a relational, emergent property of learning ecologies rather than a static feature of the environment. Work by Rietveld and Kiverstein (2014) and Chemero (2009) has emphasized the dynamic coupling of perception, action, and sociocultural norms, while Larsen-Freeman (2018) and Tomasello (2019) have shown that language development unfolds through iterative interactions that blur the line between environment and cognition.

Within this international conversation, Nguyen (2022a,b, 2025a,b) and Nguyen and Doan (2025a,b) extend the concept by theorizing affordance as a multi-layered neuro-ecological network in which perceptual salience, learning valence, normative constraints, and intentional agency interact with technological mediation. Their Five-Dimensional Affordance Framework (perceptibility, valence, compositionality, normativity, intentionality) resonates with Rietveld and Kiverstein's “skilled intentionality” hypothesis while adding an explicit focus on AI-supported learning environments and learner-driven reflection. Idiographic case analyses of AI-mediated interaction (as suggested in Nguyen, 2025b) further illustrate how individual learners notice, revalue, and orchestrate affordances across time, a process that aligns with usage-based views of emergent grammar (Ellis, 2008) and with research on self-regulated engagement in technology-rich settings (Godwin-Jones, 2019). Together, these strands suggest that affordances in language education are understood as co-constructed opportunities for action and meaning-making that recruit neural, social, and technological resources, rather than as teacher-controlled inputs to tune to a notional i+1 finely.

This ecological view holds that language learning is fundamentally relational (van Lier, 2004; Larsen-Freeman, 2018). It is about the learner in context, continuously adapting to and co-creating their environment. In such a view, simplified input (the typical implementation of i+1) appears impoverished. Comprehensibility is achieved not only by simplifying linguistic forms but also by providing meaningful context and multimodal clues in the environment (Ellis, 2008; van Lier, 2004; Tomasello, 2019). For example, caretaker speech to children (often cited by Krashen as evidence for the necessity of simplified input) is linguistically simpler and highly contextualized, about the here-and-now with shared situational cues (Snow, 1977; Tomasello, 2019). Research has shown that even in first language acquisition, caretaker or “motherese” speech is not uniformly simple in structure; parents may use complex sentences, but communication remains comprehensible due to gestures, intonation, and immediate context (Snow, 1977; Tomasello, 2019). Critics of the Input Hypothesis pointed out that “comprehensible input” does not necessarily mean “linguistically simplified” input (White, 1987; Gregg, 1984; Ellis, 2008). Instead, input can be made understandable through contextual support. For instance, a teacher could use a normal (even complex) sentence in the target language, accompanied by a picture, demonstration, or facial expressions, so learners can grasp the meaning. The CI hypothesis did not adequately acknowledge this distinction; it tended to conflate comprehensible input with simplified input. White (1987) and Gregg (1984) noted that an overemphasis on simplification can be misguided. If input is too sterilized and simplified, learners may be deprived of exposure to authentic language features and challenges that prompt development. In an ecological approach, by contrast, the goal is not to simplify the language artificially but to amplify the environment and provide a rich semiotic budget that the learner can draw on (van Lier, 2004). This could mean diverse sources of input (visual, auditory, textual), interactive opportunities, and meaningful tasks that jointly make complex language accessible.

Another key concept in ecological theory is agency, as learners are agents who actively explore and exploit their environment (van Lier, 2004; Larsen-Freeman, 2018). They are not passive recipients of input; they seek out information, respond to feedback, and initiate communication. The environment “affords” different actions to those looking for them. For example, in a classroom rich with print (posters, labels) and social interaction, an engaged learner might notice a new written word (an affordance for reading), ask a classmate what it means (turning it into an affordance for dialogue), and then use it in a project (an affordance for production). None of these steps is driven solely by externally provided input labeled i+1; they are driven by the learner's interaction with a multifaceted environment (van Lier, 2004). Van Lier emphasized the “centrality of interaction” in affordances, noting that “affordances consist in the opportunities for interaction that things in the environment possess relative to the sensorimotor capacities of the animal” (van Lier, 2004, p. 92). In language terms, any given utterance or object might afford different things to different learners. One notices a grammatical form they have almost acquired (i+1 for grammar), another gets the primary meaning (i+0 for comprehension), and another perhaps finds it too difficult (i+ TooHigh) but picks up a non-verbal cue instead. The rigid notion of i+1 as a precise incremental step is incompatible with this fluid, learner-centered view. Instead of an i+1 input, ecological theory talks about calibrating tasks and contexts to be challenging yet manageable, and about learners adapting their attention to whatever input or cues are salient and valuable at the moment (Larsen-Freeman, 2018; Ellis, 2008).

Ecological perspectives also highlight the importance of multimodality and authenticity. Language in the real world is typically embedded in a multimodal context; gestures, tone of voice, facial expressions, physical surroundings, and cultural cues all provide meaning (Gullberg, 2010; van Lier, 2004; Tomasello, 2019). Research on multimodal learning shows that adult learners benefit from face-to-face interaction with all these cues, allowing them to leverage non-linguistic information to comprehend and acquire language (Gullberg, 2010; Muntaha et al., 2023). Thus, comprehension can often be achieved without simplifying language by offering multiple modes of understanding. For instance, learners might not know a word, but seeing the speaker's pointing gesture or the object in question allows them to infer it. This process differs significantly from the CI notion of a teacher intentionally using a word just slightly above the students' level. It is more organic and learner-driven; the learner encounters richer input, making it comprehensible through contextual inferencing. Ecological language learning theory argues that such rich, contextualized experiences enable deeper learning, mirroring how language is used and learned outside the classroom (van Lier, 2004; Tomasello, 2019; Lai et al., 2015). Simplified input in a sanitized classroom, by contrast, might be less effective because it strips away the very affordances (context, interaction, multisensory cues) that learners need to make form-meaning connections.

Moreover, the ecological approach considers the broader environment of the learner, including out-of-class exposure, community, technology, and social networks (Lai et al., 2015). In today's world, language learners often have access to a vast environment beyond the teacher's input, from online media and social platforms to multicultural peer groups. Ecological research on out-of-school learning and technology integration suggests that the most successful learning happens when learners can navigate dynamic, unpredictable contexts and treat challenges as affordances for growth (Lai et al., 2015; Godwin-Jones, 2019). For example, a student might play a video game in the target language and initially understand little (far beyond i+1), but the game's visuals and feedback loop gradually make more language comprehensible. They acquire some phrases not because someone finely tuned them to the student's level, but because the ecological system of player-goal-feedback in the game encouraged repeated exposure, hypothesis-testing, and social interaction with other players. This kind of learning scenario is invisible in the CI framework, yet increasingly common and powerful in reality.

An ecological perspective critiques the Comprehensible Input hypothesis because learning is contextually situated, not a mere input-output process (Larsen-Freeman, 2018; van Lier, 2004). Language emerges from meaningful activity in authentic environments, where learners actively use environmental resources (including other people) to progress. Krashen's i+1 concept isolates language from its environment, implying that one can feed language at the right level into a learner, and acquisition will happen. Ecological theory counter-argues that what matters is engaging learners in rich interactions (affordances) that naturally contain the ingredients for learning, whether or not they are at a perfectly calibrated difficulty level. Indeed, Brown (2000) noted that input should not be so far beyond learners as to overwhelm them, but also “not so close to their current stage that they are not challenged at all” (p. 277), advocating a balanced difficulty, not a precise one-level step. The challenge and support come from the environment as a whole, not just linguistic tweaking. Therefore, from an ecological standpoint, the Comprehensible Input hypothesis is both conceptually and practically limited: conceptually, it misses the relational, interactive essence of learning; practically, it can lead to dull, contrived pedagogy that fails to exploit the whole ecology of learning opportunities available in and out of the classroom.

## Personalized, adaptive learning in the twenty first century vs. the i+1 hypothesis

### The i+1 formula for individuals: from enlightenment to doubt

Krashen's i+1 formula, the idea of always aiming input just one notch above the learner's current competence, was an elegant pedagogical heuristic in the 1980s. However, as a model for guiding instruction, i+1 has severe limitations, especially considering modern personalized learning approaches. One major issue is scalability and individualization: in a classroom of diverse learners (or in self-study scenarios), how can one determine and deliver the optimal i+1 input for everyone? Krashen acknowledged that finely tuning input is ideal but offered no concrete mechanism for assessing each learner's input on the fly (McLaughlin, 1987; Ellis, 2008). In practice, teachers use broadly simplified language or textbook scripts labeled by level, a one-size-fits-all approach that may be i+5 for one student and i−1 for another. Measuring “current level” is itself problematic, as McLaughlin (1987) pointed out; language proficiency is not a single number, and learners have spiky profiles (firm in some areas, weaker in others). Krashen's theory assumes a homogeneous, linear progression (stage 4 to stage 5, etc.), yet research shows that learners often acquire different parts of language along different trajectories. For example, a learner might have a high level of listening comprehension but a lower level of grammar accuracy; what is i+1 in this case? The rigidity of the concept fails to accommodate these realities. Brown (2000) noted “the indeterminacy of the sequences” in language learning and argued that attempts to describe i+1 precisely “will practically lead us nowhere” (p. 277).

### The challenge of individualization and scalability

In contrast, twenty first century personalized learning models aim to dynamically adapt to each learner's needs in a way far beyond the static i+1 formula. Contemporary personalized learning theories such as rhizomatic learning (Lian, 2004, 2011; Lian and Sangarun, 2023) and affordance-based language learning (Nguyen, 2022a,b) reject linearity, advocating instead for non-hierarchical, multidirectional pathways of language growth, where learning emerges from complex, dynamic connections among experiences, resources, and participants. With the advent of educational technology, big data, and artificial intelligence, it has become feasible to do what human teachers cannot easily do: continuously assess a learner's performance and adjust content difficulty in real time (Godwin-Jones, 2019; Paladines and Ramirez, 2020; Lin et al., 2023). These AI-powered adaptive learning systems highlight how simplistic the i+1 concept now appears. For instance, modern language learning platforms (such as Duolingo or Babbel) employ algorithms to estimate a user's proficiency on various micro-skills and then select or generate practice items accordingly (Paladines and Ramirez, 2020; Lin et al., 2023). Recent data-driven personalized learning models integrate big data analytics to tailor foreign language learning paths to individuals, using real-time analysis of learner responses to provide practice that is neither too easy nor too hard, but optimally challenging for that particular learner at that moment (Xia et al., 2024; Paladines and Ramirez, 2020; Lin et al., 2023). The results have been impressive: in case studies, classes using such adaptive systems saw significant improvements in student engagement and comprehension, with data showing faster mastery of language concepts than traditional methods (Paladines and Ramirez, 2020; Lin et al., 2023).

Another drawback of i+1 is that it implicitly assumes the teacher (or material designer) knows best what the learner is ready for next. It is a top-down approach, rooted in syllabus design (like structural grading of textbooks) (Nguyen, 2022b). However, language learning is not strictly linear, and oftentimes learners benefit from spaced repetition, recycling, and revisiting simpler material even as they tackle harder input (Brown, 2000; Ellis, 2008). The i+1 formula offers no room for such pedagogical strategies; it is only forward-moving. In practice, good teachers often depart from strict i+1 progression to review or to address gaps (which Krashen might say is an unnecessary “learning” activity, but research says otherwise). Meanwhile, adaptive learning systems naturally incorporate review because they detect when a learner falters on something and can represent easier examples or provide hints. They also can adjust pacing: some learners might leap from i to i+3 if they show rapid mastery (skipping intermediate steps), whereas others might need many micro-steps. I+1 as a fixed step size is too crude for this nuance.

Scaling up to large classes or diverse populations further exposes i+1′s limitations. In a class of 30 students, each has a different i. A teacher speaking in one register will inevitably be incomprehensible to some (i+TooHigh) and boringly easy to others (i+0 or lower). Krashen's advice here was that if input is incomprehensible, add more visuals or simplify until it is understood (Krashen, 1985). Nevertheless, simplifying to the lowest common denominator can hold back advanced learners, while using richer language to challenge them might lose the beginners. This is a perennial dilemma in mixed-ability teaching. Modern solutions emphasize differentiation, providing multiple streams of input or tasks at varying levels, often with the help of technology or collaborative grouping (Ellis, 2008; Paladines and Ramirez, 2020; Lin et al., 2023). For example, some teachers use online reading platforms that adjust the text complexity for each student while covering the same content, or AI tutoring chatbots that students can interact with at their own level, getting personalized feedback. These approaches align with what an adaptive, individualized learning path would prescribe, and they have shown improved outcomes in both language and other subjects (Godwin-Jones, 2019; Paladines and Ramirez, 2020; Lin et al., 2023). By contrast, a teacher strictly adhering to comprehensible input might limit all discourse to a narrow band of vocabulary and grammar (the presumed i+1 for the class), potentially under-stimulating many and still confusing some. Over time, this could result in fossilization or boredom. The one-level-above approach does not scale well to diverse needs, whereas intelligent systems or flexible pedagogies can cope with variability.

At this point, it is of paramount importance to present combined insights from neurolinguistics and ecological psychology to support a view of language learning, including different complex stages, rather than just the artificial dichotomies of learning and acquisition, as a process of continual coordination between the learner's neural systems and the environment where learning takes place. Neurolinguistic research demonstrates that experience-dependent plasticity, interaction, feedback, and embodied activity contribute to the neural changes that sustain language learning. Ecological scholarship shows that learners perceive and act on affordances, the opportunities for action and meaning that emerge within social and material settings. Taken together, these findings define a neuro-ecological perspective in which language development reflects the gradual alignment of neural adaptation with environmental affordances. In this view, repeated interaction and feedback strengthen neural circuits while learners simultaneously detect and exploit the social, material, and technological opportunities for action that the environment offers. As neural representations become more efficient, learners can perceive finer distinctions in the environment, which in turn invites richer interaction and further neural change. This theoretical integration provides the foundation for the empirical data presented in the next section.

## Empirical support and research critique in the age of personalization

Following the theoretical arguments of the previous part, the following section seeks to provide empirical studies for the adoption of another perspective on language learning. At the point this article is written, Krashen's i+1 concept has never been fully validated empirically in the same way that modern adaptive learning algorithms are tested through A/B experiments and large-scale learning analytics. It remains a theoretical proposal derived from observation rather than a falsifiable model. Studies attempting to show that input exactly at i+1 produces superior learning have yielded mixed results. For example, Namaziandost et al. (2019) compared Iranian EFL learners exposed to i+1, i-1, and mixed-difficulty input and found that while i+1 groups improved reading comprehension and motivation, the advantage over mixed input was not decisive. Research on form-focused instruction likewise shows that a balanced combination of input types often yields the most tremendous gains. Ghorbani and Atai (2012) reported that explicit form-focused instruction led to more substantial gains in both explicit and implicit grammar knowledge than purely implicit treatment. A large meta-analysis by Spada and Tomita (2010) confirmed that explicit instruction produces significant accuracy benefits, especially for more complex language features, and that a mix of implicit and explicit activities can optimize fluency and confidence. Norris and Ortega (2000) seminal meta-analysis likewise demonstrated robust overall effects of instruction on L2 grammatical accuracy, with explicit approaches outperforming input-only exposure. These findings align with contemporary data-driven approaches in which learning analytics reveal individualized patterns of difficulty. For instance, an AI tutor can detect that a learner retains vocabulary best when new words are introduced in a moderately challenging context (perhaps beyond i+1) and then recycled in easier contexts. Such adaptive, mixed-difficulty sequencing is now standard in intelligent tutoring systems, whereas the Input Hypothesis remains static and offers no mechanism for personalized adjustment.

The rise of AI-supported learning environments provides a direct counterpoint to the comprehensible classroom input. In an AI language tutor scenario, the system can play multiple roles: conversational partner (providing interactive input and output practice), assessor (gauging the learner's responses), and assistant (offering hints, translations, or simpler rephrasings as needed). This dynamic interplay enables personalized and adaptive support, fundamentally different from Krashen's one-way input model (Paladines and Ramirez, 2020; Lin et al., 2023). Whereas comprehensible input, as initially conceived, was largely unidirectional (teacher talks, student listens), AI tutors respond to the learner in real time. Interactive, responsive systems inherently personalize the experience, ensuring the learner is neither bored nor lost for long (Godwin-Jones, 2019; Paladines and Ramirez, 2020; Lin et al., 2023). Early research indicates that such systems improve learning outcomes and greater learner autonomy (Godwin-Jones, 2019; Paladines and Ramirez, 2020; Lin et al., 2023). As we incorporate these technologies, the notion of a fixed “i+1” fades away, replaced by the concept of a continuously calibrated zone for each learner, responsive to their unique progress and needs.

In sum, while i+1 was a helpful reminder not to overwhelm learners with incomprehensible input, it is an oversimplified and outdated guide for instruction, as there is very limited research to validate this hypothesis until now. Also, it fails to account for individual differences, the non-linearity of language learning, and the proven efficacy of adaptive, interactive pedagogies. Modern personalized learning paradigms, informed by AI and data, offer a more powerful and precise toolkit to achieve what i+1 aspired to: keeping learners in that productive, challenged-but-comprehending zone. As such technologies and approaches gain prominence, clinging to the simplistic i+1 formula becomes unnecessary and a hindrance to language teaching innovation.

## Empirical research in three decades: evidence for a neuro-ecological approach

On the other hand, a growing body of empirical research strongly and comprehensively supports the theoretical critiques above in the past decade. Studies in neurolinguistics, ecological second language learning, and adaptive learning conclude that effective language learning involves much more than understanding level-appropriate input. This article highlights key findings from recent research that reinforce the shortcomings of the Comprehensible Input hypothesis and point toward more comprehensive frameworks.

### Neurolinguistic and cognitive evidence

Multiple recent studies conclude that social interaction, embodiment, and feedback drive measurable neurocognitive change in second language learning. Li and Jeong (2020), synthesizing research across child language, adult SLA, and cognitive science, show that interactive learning produces enhanced behavioral outcomes and increased functional connectivity in brain language networks, whereas non-interactive listening does not. One experiment they reviewed found that adults who engaged in face-to-face interactive sessions, complete with turn-taking and gestures, showed stronger *post-test* gains in speaking and comprehension and greater connectivity in language-related neural networks than peers who spent the same amount of time on non-interactive listening. Reggin et al. (2023) likewise provide consensus evidence for embodied language learning, demonstrating that sensorimotor engagement and affective resonance facilitate retention and transfer of new forms. They review studies in which children who learned action verbs through physical enactment (e.g., learning push by actually pushing) retained and generalized those words better than children who learned through static definitions, and they note similar embodied effects in adult learners.

Another example is the rapidly expanding body of research that offers convergent neurolinguistic evidence using Verbotonal-based methods. Experiments with Chinese EFL learners show that dichotic listening and filtered/unfiltered input manipulation can induce measurable shifts in cerebral lateralization for speech processing (Lian et al., 2020), a neural marker of phonological learning that signals reorganization of auditory pathways. Training that combines filtered auditory input with rhythmic body movement and immediate feedback, as in Yang et al. (2023), stimulates auditory—motor integration networks (superior temporal gyrus, premotor cortex), directly supporting the perception—action loops emphasized in embodied cognition. Complementary studies demonstrate the role of corrective feedback: Li and Lian (2022) and Wen et al. (2020) document significant improvements in English intonation and pronunciation when learners receive targeted corrective feedback during training, while Liu et al. (2021) and Guo et al. (2020) show that web-based autonomous listening systems promote self-regulation and adaptive engagement. Such tasks recruit executive functions (working memory, attention shifting, inhibitory control) that predict long-term L2 attainment. Complementing these findings, Casillas (2020) tracked absolute-beginner English speakers in a 7-week domestic immersion program and found robust reductions in Spanish stop voice-onset-time (VOT) after only 21 days of exclusive L2 use. Bayesian growth-curve analyses showed non-linear, segment-specific learning trajectories, providing direct evidence that new phonetic categories can emerge rapidly under high-input, interaction-rich conditions, precisely the type of brain—environment coupling predicted by neuro-ecological models.

These findings align with a large body of interactional-feedback research. Meta-analyses show that learners who receive recasts, prompts, or other interactional feedback make significantly greater gains in grammatical accuracy than those who do not (Li, 2010; Lyster and Saito, 2010). Neurolinguistic studies using EEG reveal Error-Related Negativity (ERN) and P600 responses when learners encounter corrective feedback, indicating that the brain is actively updating linguistic representations (Morgan-Short et al., 2012). Therefore, the behavioral improvements reported in the Chinese Verbotonal studies imply underlying neural adjustments consistent with these ERP signatures. This multi-regional evidence demonstrates that language development is not a process of passive input absorption but a brain—body—environment coupling in which social interaction, multimodal sensory stimulation, and adaptive feedback jointly drive neuroplastic change. Far from supporting a static Input Hypothesis, these findings show that optimized auditory signals, sensorimotor engagement, and feedback loops, whether delivered face-to-face or through AI-enhanced CALL platforms, produce neural and behavioral benefits that comprehensible input alone cannot match.

### Ecological and affordance-based research

In language education, there has been an apparent uptick in studies adopting ecological frameworks to address modern learning contexts. Lai (2015) and Rokita-Jaskow (2024) provide an overview of recent research trends from an ecological perspective, highlighting topics like the spread of technology, out-of-school learning, and linguistically diverse classrooms. They argue that ecological approaches are well-suited to describing current language education, characterized by dynamic change and unpredictability. For example, one strand of research examines informal digital language learning, how learners pick up languages through social media, online gaming, or YouTube (Sockett, 2014; Lai and Zheng, 2018). These studies often find that learners acquire a great deal incidentally by navigating these environments, leveraging affordances like subtitles, translations, peer assistance, and multimedia cues. In such settings, the input is not graded to i+1 at all; it can range from very easy to far beyond the learner's level. However, learners acquire phrases and even complex constructions because they engage with meaningful content and context. This supports the ecological claim that rich context can compensate for linguistic complexity (comprehensibility from multimodal clues rather than simplified code) and that learner agency drives learning (Lai, 2015).

An illustrative empirical study on immigrant language-minority students adapting to a new language environment found that the richness of the ecological environment (availability of community interactions, extracurricular activities, etc.) was a strong predictor of language growth (Portes and Hao, 2002). Inferentially, students with more varied affordances, e.g., joining clubs, interacting with locals, and consuming local media, progressed faster, even if much of the input was beyond their level. The researchers conclude that fostering ecological connectivity (tying classroom learning to real-life use opportunities) is key to accelerating SLA. This kind of evidence reinforces that predetermined comprehensible input, while helpful, may be neither sufficient nor as potent as immersing learners in vibrant, context-rich environments where they must actively negotiate meaning.

Another domain of ecological research is classroom interaction patterns. Studies using conversation analysis (e.g., Waring, 2016; van Lier, 2004) show that when teachers shift from monologic input delivery to dialogic interaction, student language production and uptake increase. Even small changes, like asking more open-ended questions or allowing students to control topics, create more affordances for language use. Empirical evidence from these studies indicates that classes with high interactional involvement see better vocabulary acquisition and pragmatic development than classes where the teacher does most of the talking (even when the teacher's talk is “comprehensible”). Learners benefit from being protagonists in the language environment rather than just an audience.

### Personalized and adaptive learning research

The integration of AI in language education has also yielded empirical studies that measure learning gains with and without adaptive personalization. One recent review by Xu (2024) highlights numerous instances where AI-driven systems improved learning outcomes. For example, an adaptive vocabulary learning app that used machine learning through speech recognition to predict which words an individual learner was likely to forget, and scheduled reviews accordingly, led to higher long-term retention than a non-adaptive, one-size-fits-all word list approach (Wilschut et al., 2024). Another study compared a chatbot-facilitated project-based learning (where the chatbot adjusted its language complexity based on the learner's responses) to a static script conversation; learners using the adaptive chatbot showed greater increases in autonomy and transformative changes in efficacy (Nguyen and Doan, 2025b). These findings directly support the argument that individualized pathways outperform the fixed incremental approach. Moreover, research in Intelligent Tutoring Systems (ITS) for language learning has demonstrated that students using an ITS that gives immediate, personalized learning feedback (essentially providing comprehensible output in context) learn specific linguistic constructions faster than those who only receive additional input exposure to those constructions (Godwin-Jones, 2019). This aligns with the idea that hypothesis testing and feedback (enabled by adaptive systems) accelerate learning, a dynamic absent from pure input-based methods.

Finally, data analyses from large language-learning platforms provide quantitative backing for adaptive learning. For example, a Duolingo study analyzing millions of learner interactions found that users who completed the full beginner and intermediate English courses achieved STAMP 4S proficiency scores at or above CEFR A2 benchmarks, often exceeding expectations for reading and listening (Jiang et al., 2024). Other research leveraging billions of Duolingo activity logs has shown that machine-learning models can predict which items an individual learner is most likely to forget and schedule reviews accordingly, enabling more efficient retention than static word lists (Portnoff et al., 2021; Cui and Sachan, 2023). Another independent evaluation of Duolingo's Spanish for English speakers course by Vesselinov and Grego (2012) reported that beginners required about 34 h of Duolingo study to cover the material of one semester of U.S. college Spanish. In a separate large-scale analysis of learner interaction logs, Settles and Meeder (2016) estimated an average gain of 8.1 WebCAPE points per hour of Duolingo study. These findings apply specifically to beginning learners of Spanish under the testing conditions of those studies and should not be interpreted as a direct, randomized comparison with traditional classroom instruction. This kind of individual optimization goes well beyond i+1. Students might get i+0 repetition on one concept they struggle with, i+2 stretch on another concept they grasp quickly, and even 1 on something forgotten, all determined by their interaction data. Notably, the personalized system often gives users material that is below or at their level for review, and occasionally jumps ahead to challenge them on upcoming material if they are doing well. This mix was more effective than strictly maintaining an i+1 cadence. Such real-world data at scale makes a compelling case that adaptive algorithms can learn more efficiently than static input staging.

In summary, both theoretical developments and empirical research of the last decade comprehensively support a shift away from the narrow Comprehensible Input paradigm toward a more integrative, neuro-ecological approach. This approach recognizes that:

(1) Active engagement and social interaction yield better neural and learning outcomes than passive input alone,

(2) Embodied, context-rich learning leads to deeper learning than disembodied, simplified input,

(3) Learners thrive on affordances in a rich environment, even if the input is not perfectly leveled, and

(4) Personalized, adaptive instruction outperforms one-size-fits-all input delivery by tailoring challenges and support to individual needs.

Together, these findings validate the critiques of Krashen's hypothesis and provide the foundation for new neuro-ecological paradigms in language education.

## Conclusion: toward a neuro-ecological framework for language education

Four decades after Krashen's Comprehensible Input hypothesis, the field of language education stands at a crossroads shaped by advances in brain science, a richer appreciation of context and interaction, and the emergence of powerful technological tools. In this new era, persisting with the CI hypothesis as the primary compass is neither conceptually sufficient nor empirically justified. Rather than viewing language learning as a linear, input-driven process, current evidence and theory compel us to embrace frameworks that account for acquisition's dynamic, interconnected, and personalized nature.

Affordance-based learning foregrounds the opportunities for action in learners' material, social, and technological environments. In this model, language development arises through learners' active engagement with rich, multimodal contexts that provide many meaningful cues and possibilities for communication. The teacher's role shifts from input transmitter to designer of environments, scaffolding, and tasks that encourage learners to perceive, exploit, and even create new affordances. Besides, connectivism (Siemens, 2005) situates knowledge within networks of people, artifacts, and digital resources. Language learners today navigate a complex web of social and technological connections, learning not only from teachers and textbooks but from global communities, AI-driven platforms, and participatory digital cultures. It is important to note that Connectivism may have overstated its claim in suggesting that knowledge can be stored in networks, as digital systems today primarily store and process information rather than embody knowledge. Nevertheless, the rapid development of artificial intelligence suggests a possible future in which machines and socio-technical systems may increasingly approximate or even participate in processes of “knowing.” Effective language education now means equipping learners with the skills to construct, curate, and traverse these networks, becoming autonomous agents in their own learning trajectories. Additionally, rhizomatic learning (Lian, 2004, 2011; Lian and Sangarun, 2023) offers a non-linear, non-hierarchical metaphor for knowledge growth. In this view, language development resembles a rhizome's sprawling, interconnected roots, unpredictable, emergent, and uniquely individualized. Learning pathways are forged through exploration, adaptation, and continual reconfiguration, rather than predetermined sequences.

Bringing these models together, underpinned by advances in neuroscience, cognitive science, and educational technology, moves us decisively beyond the limitations of Comprehensible Input. Modern language classrooms can harness adaptive platforms, AI tutors, immersive digital environments, and authentic, multimodal resources, allowing each learner's needs, interests, and context to shape their experience. The neuro-ecological language learning models we have implemented so far, such as ChatGPT-mediated self-regulated learning (Nguyen and Doan, 2025b), idiographic affordance-uptake tracking (Nguyen and Doan, 2025a), and verbotonal–CALL integrations that optimize auditory input and corrective feedback, represent only the earliest generation of brain—body—environment designs. These projects already display the key features of a neuro-ecology: learners engage in real-time prediction and error monitoring, adaptive algorithms fine-tune input, and affordances emerge from the interaction of cognitive, social, and technological forces. Yet these systems remain bounded by current platforms, institutional curricula, and human facilitation, functioning as carefully curated pilots rather than fully self-organizing ecologies. Looking ahead, advances in large-scale sensing, generative AI, and decentralized networks are poised to move beyond today's adaptive tutors toward emergent learning ecosystems in which affordances are not merely detected but dynamically co-created and recombined across distributed communities. In such environments, AI agents may act as co-learners that negotiate goals, generate novel action possibilities, and evolve alongside human participants, while learners continuously recalibrate norms, values, and strategies. The Five-Dimensional Affordance Framework, including perceptibility, learning valence, compositionality, normativity, and intentionality (as suggested in Nguyen, 2025a,b), provides a critical analytic lens for studying these next-generation systems, ensuring that future designs remain grounded in the relational dynamics of brain-body-environment coupling rather than regressing to static models of input.

**Figure 1:**
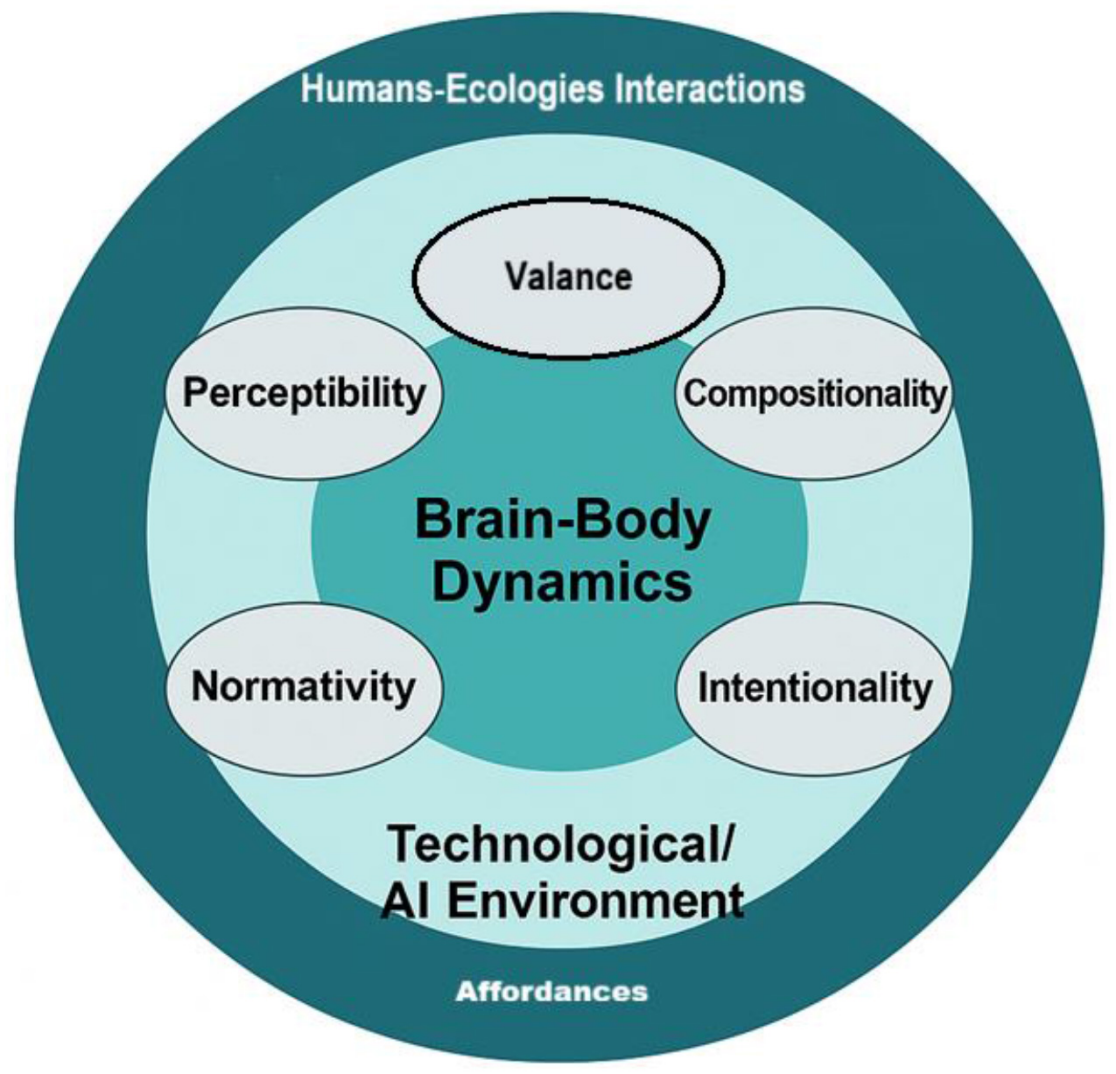
Neuro-ecological learning umwelt.

The task for educators and researchers is to design and facilitate learning ecologies that are rich in affordances, networked in their resources, and rhizomatic in their flexibility. Learners are not passive consumers of input, but active participants, explorers, and creators, within an ever-evolving landscape of linguistic possibility with fine-tuned neurological support from the technological/AI environment to enhance humans-ecologies perception and interactions as demonstrated in Figure 1. It is important to note that these interactions can be highly complex, adaptive, emergent, and sophisticated in reality. By embracing these contemporary paradigms, language education becomes more relevant, practical, and empowering, preparing learners for the complexities of communication in a rapidly changing world.
